# The Compassionate Engagement and Action Scale for Youths: psychometric properties in a clinical psychiatric Swedish sample

**DOI:** 10.3389/fpsyg.2025.1653979

**Published:** 2026-01-05

**Authors:** Linda Wallin, Carl-Göran Svedin, Marie Wiberg, Inga Dennhag

**Affiliations:** 1 Department of Clinical Science, Child and Adolescent Psychiatry, Umeå University, Umeå, Sweden; 2 Department of Social Work, Marie Cederschiöld University, Stockholm, Sweden; 3 Department of Statistics, Umeå School of Business, Economics and Statistics, Umeå University, Umeå, Sweden

**Keywords:** compassion, psycometrics, reliability, validity, confirmatory factor analysis, clinical youth sample

## Abstract

**Background:**

Compassion contributes to wellbeing and serves as a protective factor against mental health problems in young people. The Compassionate Engagement and Action Scale for Youth—Swedish version (CEASY-SE) is a self-report questionnaire that assesses compassion across six competencies, organized into three scales: Self-Compassion, Compassion for Others, and Compassion from Others. While previously validated in a Swedish school sample, its applicability in clinical psychiatric populations remains unexplored. This study aimed to evaluate the psychometric properties of CEASY-SE in a clinical psychiatric sample of Swedish youth aged 16–22.

**Methods:**

A cross-sectional study was conducted, collecting self-reported data from young people (*N* = 355) receiving care in child and adolescent psychiatry and primary care. We assessed the CEASY-SE's factor structure, reliability, and validity (internal consistency, convergent, divergent, construct, and criterion-related validity). Sex and age differences were also analyzed, along with comparisons of total scores across diagnostic groups.

**Results:**

Confirmatory factor analyses supported a two-factor model within each of the three scales. Internal consistency was good to excellent across all scales (α ranging from 0.75 to 0.92), except for the Self-Compassion Engagement subscale among males (α = 0.62; ω = 0.60). Convergent and divergent validity were satisfactory. Among the three CEASY-SE scales, Self-Compassion showed the strongest correlations with mental health outcomes. Sex differences primarily affected Compassion for Others, and a sex-by-age interaction was found for both Compassion for Others and Self-Compassion. A total score of 40 was associated with an 8.41-fold increase in the predicted probability of depression symptoms compared to a score of 100. The lowest scores were found among patients with eating disorders and depression.

**Conclusions:**

The CEASY-SE demonstrates acceptable to excellent psychometric properties in a clinical psychiatric sample of youth. It is a promising tool for clinicians and researchers to assess and promote compassion in young people, with potential relevance for interventions aimed at improving mental health outcomes.

## 1 Introduction

Mental health problems among young people are a growing global concern, with one in seven experiencing a mental disorder (World Health Organization, 2024). These difficulties often arise during critical developmental periods and are associated with increased self-criticism, perfectionism, and social comparison, which in turn contribute to psychological distress and impede recovery (Etherson et al., 2022). Compassion-focused therapy (CFT) addresses these maintaining processes by cultivating a compassionate inner voice to counter shame and self-criticism.

Despite the increasing interest in compassion-based approaches for youth, research remains limited, particularly in clinical populations. To address this gap, the Compassionate Engagement and Action Scale for Youth—Swedish version (CEASY-SE) was developed as an adaptation of the adult Compassionate Engagement and Action Scale (CEAS) (Gilbert et al., 2017). The CEASY-SE assesses compassion across three relational dimensions: compassion for others, compassion from others, and self-compassion. The scale captures both engagement with suffering and compassionate action, providing a comprehensive framework for understanding how compassion is experienced and expressed in youth. While the CEASY-SE has shown good to excellent psychometric properties in a Swedish school sample (Henje et al., 2020), its applicability in clinical psychiatric populations has yet to be examined.

Although studies suggest that self-compassion is associated with better emotional wellbeing in youth (Bluth et al., 2018; Egan et al., 2022; Lathren et al., 2019), findings on developmental trajectories are mixed. Some studies report a decline in self-compassion during early adolescence, particularly among girls (Bluth et al., 2017), while others suggest an increase in late adolescence (Tavares et al., 2024). Compared to adults, adolescents may also experience greater fluctuations in compassion-related processes due to ongoing identity formation and the development of emotional regulation (Gilbert, 2017; Cunha et al., 2023). These developmental dynamics underscore the need for age-appropriate tools to assess compassion in youth, particularly in clinical settings.

Compassion is a multidimensional construct, shaped by individual and cultural factors (Jiang et al., 2023; Sinclair et al., 2022; Strauss et al., 2016). A recent scoping review identified 36 compassion-related measures (Jiang et al., 2023), but only two, Compassion Motivation and Action Scales (CMAS) (Steindl et al., 2021) and CEAS (Gilbert et al., 2017), capture all three relational dimensions. Among these, CEAS is more widely validated and emphasizes compassionate action, making it a strong foundation for youth adaptation.

In this study, compassion is defined as “sensitivity to suffering in self and others with a commitment to alleviate and prevent it” (Gilbert et al., 2017). This conceptualization is grounded in evolutionary psychology, which views compassion as part of the caregiving system that evolved to promote social bonding and survival (Gilbert, 2017). Unlike empathy and kindness, compassion includes a motivational component to act in response to suffering, directed both inward and outward (Gilbert, 2016, 2005, 2020). This evolutionary perspective provides a theoretical foundation for the CEASY-SE, which captures both the emotional engagement with suffering and the actions taken to alleviate it.

Validating CEASY-SE in a clinical psychiatric sample is essential to ensure its reliability and applicability in mental health care. A validated tool would support researchers and clinicians in assessing compassion-related processes and tailoring interventions for young people. It would also contribute to the growing field of compassion-based interventions by enabling more precise measurement of therapeutic outcomes.

This study aims to validate the CEASY-SE in a clinical psychiatric sample of Swedish youth aged 16–22. We examine its psychometric properties, including factor structure, reliability, and validity, to determine its utility in clinical practice and research.

## 2 Methods

This study employed a cross-sectional design conducted in northern Sweden between October 2018 and August 2021 as a part of the research project “*Adolescents' Experience of Mental Illness - Psychometric Properties of New Swedish Versions of the Test (UPOP)”*(Dennhag, 2018).

The study adhered to established guidelines for the psychometric validation of psychological instruments (Ayearst and Bagby, 2010). This included confirmatory factor analysis to assess dimensionality, internal consistency estimates using both Cronbach's alpha and McDonald's omega, and multiple forms of validity testing (convergent, divergent, construct, and criterion-related). Additionally, we examined demographic effects and controlled for potential confounders, ensuring a comprehensive evaluation of the CEASY-SE in a clinical psychiatric sample of youth.

Ethical approval was granted by the Regional Ethical Review Board in Umeå, Sweden (number 2018/59-31). All procedures adhered to ethical standards to ensure participant protection and data integrity.

### 2.1 Procedure

The CEASY-SE was translated into Swedish with permission from the original authors of the CEAS (Gilbert et al., 2017). A back-translation method was used to ensure semantic and content equivalence in cross-cultural research (Hambleton, 2004). Henje et al. (2020) provide a detailed description of the translation procedure.

Participants were recruited from four child and adolescent psychiatric clinics, one primary care youth clinic, and one general primary care health clinic in four towns in northern Sweden. After approval from the operational managers, information flyers were placed in the clinics' waiting rooms. Additionally, patients with self-reported anxiety and depression, who were receiving care at the clinics, received study invitations via SMS or mail. Research assistants contacted both responders and non-responders by telephone to provide comprehensive verbal and written information about the study. This included details about the study's purpose, procedures, potential risks, benefits, and the option for voluntary participation. Informed consent was obtained from all participants, with additional consent from caregivers for those under 18 years of age. The first 65 participants completed paper-based assessments between October 2018 and May 2020. Between May 2020 and August 2021, 290 participants were invited to complete online assessments. Due to the impact of the COVID-19 pandemic on data collection, as well as delays in data processing, analysis, and manuscript preparation, the completion of final analyses and manuscript preparation occurred later than initially planned.

Participants were asked if they received mental health care and to report any current diagnoses. For those recruited from psychiatric clinics, diagnoses were extracted from medical records by a research assistant after data collection. Diagnoses were classified according to the Diagnostic and Statistical Manual of Mental Disorders, Fifth Edition (DSM-5) (American Psychiatric Association, 2013) or the International Statistical Classification of Diseases and Related Health Problems, 10th Revision (ICD-10) (WHO, 2019), and grouped into clusters.

Confidentiality was ensured through secure data storage and anonymization. Paper-based assessments were securely stored at Umeå University. Online data were collected using Research Electronic Data Capture (REDCap) (Harris et al., 2009) hosted at Umeå University. Upon completion, participants were reimbursed with a gift card worth 200 SEK (approximately 20 EUR).

### 2.2 Participants—Psychiatric clinical sample

A total of 582 participants were invited to participate, of whom 358 (61 %) agreed. This sample size is considered adequate for psychometric evaluation in cross-sectional designs (Evers et al., 2013), and the relatively high response rate (61%) enhances the generalizability of the findings. Attrition (*n* = 224) was primarily due to ineligibility, mostly related to age restrictions (*n* = 204; 90%). The eligibility criteria were as follows: (1) aged 16–22 years, (2) currently receiving care at one of the recruiting sites, (3) self-reported or parent-reported symptoms of depression and/or anxiety (all comorbidities were allowed), (4) for individuals with a recent history of suicide attempt or psychiatric inpatient-care (a minimum of 3 months had to have passed since the suicidal event or hospitalization discharge), and (5) fluent in written Swedish and able to complete the self-assessment.

The age range of 16 to 22 years was selected to capture a developmentally sensitive period marked by heightened emotional vulnerability. This range also aligns with the structure of Swedish youth healthcare services and with our randomized controlled trial (RCT) intervention study on compassion-focused therapy for young people (Vestin et al., 2025).

### 2.3 Comparative normative school-sample

For comparative purposes, a normative sample of Swedish young people (*N* = 316; 213 girls, 67%) aged 15–20 years (M = 17.07, SD = 1.36) was collected by our research group (Henje et al., 2020). This sample was used to contextualize the clinical findings and assess differences in compassion-related constructs between clinical and non-clinical youth populations.

### 2.4 Self-assessment measures

#### 2.4.1 Measurement for the dependent outcome variable

##### 2.4.1.1 The Compassionate Engagement and Action Scale for Youth—Swedish version (CEASY-SE)

The original CEAS was developed to assess six key competencies that support compassionate engagement and action (Gilbert et al., 2017). These competencies are divided into two main categories:

- Engagement: (1) motivation to attend to suffering, (2) attentional sensitivity and mindful attunement to distress and need, (3) sympathetic reaction (being emotionally moved), (4) tolerance of emotions arising from engaging with suffering, (5) empathic understanding of the nature of suffering, and (6) a non-judgmental attitude.
- Action: (1) engaging in helpful actions, (2) imagining scenarios, (3) reasoning, (4) behaving compassionately (which may involve acting courageously), (5) using bodily awareness to stabilize the mind, and (6) allowing appropriate emotional responses to guide action.

The CEASY-SE is a Swedish adaptation of the CEAS for youth aged 15–20 (Henje et al., 2020). It measures compassion across three dimensions/scales: (1) Compassion for Others, (2) Compassion from Others, and (3) Self-Compassion. Each scale includes nine items, totaling 27 (Henje et al., 2020) that are divided into two subscales each: (1) Engagement with distress/suffering (e.g., motivation and becoming sensitive to suffering, distress tolerance with empathic insight), and (2) Action, which focuses specifically on actions aimed at preventing and alleviating distress/suffering. Items are rated on a 10-point Likert scale (1 = never to 10 = always).

Previous psychometric evaluation of the CEASY-SE in a school sample demonstrated good to excellent internal consistency (α = 0.74–0.92) and satisfactory test-retest reliability (*r* = 0.77 for compassion for others, *r* = 0.85 for compassion for others, and *r* = 0.83 for self-compassion) (Henje et al., 2020).

#### 2.4.2 Measurements to examine convergent validity

##### 2.4.2.1 Self-Compassion scale (SCS)

The SCS (Neff, 2003) consists of 26 items assessing both compassionate (e.g., self-kindness, common humanity, and mindfulness) and uncompassionate (self-judgment, isolation, and over-identification) responses to distress. Items are rated on a 5-point Likert scale (1 = rarely, 5 = almost always). Higher item scores indicate more self-compassion. A total mean score was calculated after reversing the items on the uncompassionate subscale. The Swedish version of the SCS (Norrbottens Läns Landsting, 2013) has been validated and deemed acceptable (Nicolaidis, 2018). The scale has also been validated internationally in a large Portuguese adolescent sample, ages 12–19 (Cunha et al., 2016). In the current study, internal consistency measured by Cronbach's alpha was.88 (95% CI = [0.86, 0.90]), indicating good reliability.

##### 2.4.2.2 WHO-Five Wellbeing Index (WHO-5)

The WHO-5 (Blom et al., 2012) includes five items assessing psychological wellbeing (e.g., energy, relaxation, and interests in life). Items are rated on a 6-point Likert scale (0 = not present, 5 = constantly present), with higher scores indicating greater overall wellbeing. A total raw score (0–25) was multiplied by 4 to get a scale of 0–100, with a higher score indicating better wellbeing. This multiplication is done to make the result more intuitive and easier to interpret. The scale has been validated in a Swedish adolescent psychiatric sample, ages 14–18 (Blom et al., 2012). Internal consistency in the present study was 0.86 (95% CI = [0.84, 0.89]), indicating good reliability.

##### 2.4.2.3 Revised Child Anxiety and Depression Scale (RCADS)

The RCADS (Chorpita et al., 2005) is a 47-item self-report scale measuring symptoms of anxiety (37 items) and depression (10 items) in youth. Items are rated on a 4-point Likert scale (0 = never, 3 = always). Total sum scores for anxiety and depression were computed. The Swedish scale was obtained from the official RCADS website (Chorpita and Spence, 1998). The scale has shown good validity in Nordic samples (Esbjørn et al., 2012). In the present study, internal consistency was excellent for the anxiety subscale (α = 0.95) and very good for the depression scale (α = 0.89), indicating high reliability.

##### 2.4.2.4 Montgomery—Åsberg Depression Rating Scale—Self-report (MADRS-S)

The MADRS-S includes nine items measuring depression symptoms (Svanborg and Asberg, 1994, 2001). Items are rated on a 6-point Likert scale (0 = low, 6 = high). A total sum score was computed (range 0–54). Higher scores indicated more severe depressive symptoms. Cut-off values for symptom severity are as follows: 0–11 points indicate no depression symptoms, 12–20 points indicate mild depression symptoms, >20 points indicate moderate depression symptoms, and >40 points indicate severe depression symptoms.

MADRS-S is a reliable and valid instrument for adults. It has been validated in a Swedish adolescent psychiatric sample (*N* = 105, aged 12–17 years) with good psychometric properties and diagnostic accuracy (Ntini et al., 2020). In the current study, internal consistency was α = 0.87, indicating good reliability.

#### 2.4.3 Measurements to examine divergent validity

##### 2.4.3.1 Strengths and Difficulties Questionnaire (SDQ)

The SDQ (Hagquist, 2007) comprises 25 items that measure various aspects of difficulties and strengths. Items are rated on a 4-point Likert scale (1 = not at all, 4 = all the time), and the total score ranges from 0 to 40. In the present study, only the SDQ-impact subscale was used, which is considered a measure of global functioning. Four items (home life, friendships, learning, and leisure) were used to compute a total score (range 0–10), with higher scores indicating more significant functional impairment.

The subscale has demonstrated good validity in a Swedish adolescent sample (ages 12–18) (Hagquist, 2007). In the present study, the internal consistency was acceptable, α = 0.64 (95% CI = [0.57, 0.70]).

##### 2.4.3.2 Patient Reported Outcome Measurements Information System (PROMIS)

PROMIS includes 10 items assessing generic health outcomes in children (Cella et al., 2010, 2007). In this study we used PROMIS Pediatric Physical Activity v1.0. item bank (www.healthmeasures.net), which includes 10 items measuring physical activity levels. Most items are rated on a 5-point Likert scale ranging from 1 (no days), 2 (1 day), 3 (2–3 days), 4 (4–5 days), and 5 (6–7 days) except for one item “On a usual day, how physically active were you?”, is rated using a different scale (1 = not at all, 5 = very much). A total sum score was calculated (raw scores range 10–50), with higher scores indicating greater physical activity levels. The scale has demonstrated strong psychometric properties and validity in a Swedish adolescent sample (ages 12–19) (Rindestig et al., 2020). In the current study, the internal consistency was excellent (α = 0.92) (95% CI = [ 0.91, 0.93]), indicating high reliability.

### 2.5 Statistical analysis

All analyses were conducted using SPSS, version 29.0 (Corp, 2023), and R, version 4.4.1 (Core, 2021).

#### 2.5.1 Missing data

Three participants with > 50% missing data were excluded. Remaining variables had ≤ 1.4% missingness and were assumed to be missing at random. Expectation-Maximization was applied to the CEASY-SE scale given its suitability for psychometric data. At the same time, serial mean imputation was used for other scales to preserve sample size.

#### 2.5.2 Demographic and descriptive statistics

Demographic characteristics were summarized using frequency distribution and valid percentages. Socioeconomic status (SES) was assessed using a Swedish classification system based on six social classes (SCB, 1982).

Descriptive statistics were computed for all study variables. Normality was assessed via Kolmogorov-Smirnov and Shapiro-Wilk tests. Parametric (e.g., independent *t*-test) or non-parametric (e.g., Mann-Whitney *U*) tests were applied based on distributional assumptions.

To examine Self-Compassion scores across psychiatric diagnoses, total Self-Compassion scores were calculated for each diagnostic category. Diagnoses were categorized into a broader category, including both clinician-assigned and self-reported diagnoses. Means and standard deviations were reported for parametric comparison.

#### 2.5.3 Confounding variables

Multiple regression models controlled for confounding variables, including SES, comorbid conditions, age, and sex. Stratified analyses explored interaction effects by age and sex.

#### 2.5.4 Group differences

Independent samples *t*-tests and two-way analyses of variance (ANOVAs) were used to examine differences in compassion scores by sex (male and female) and age groups. SES associations were analyzed using Spearman's rank-order correlations, a non-parametric method robust to non-normal distribution. These analyses aimed to identify demographic patterns in compassion and to assess the CEASY-SE‘s sensitivity to subgroup variation.

#### 2.5.5 Factor analysis

Confirmatory factor analysis (CFA) was used to test the proposed factor structure of the CEASY-SE, using the Lavaan package version 0.60–3.00 BETA; (Rosseel, 2018) with diagonally weighted least squares and polychoric correlations (Koo and Li, 2016). Three CFA models were tested based on the original two-factor structure (Henje et al., 2020). Model fit was evaluated using: Satorra–Bentler Chi-square (SB χ*2*), Chi-square/degrees of freedom ratio (χ*2/df* ), acceptable if ≤ 3 (Kline, 1998), Tucker-Lewis index (TLI), comparative fit index (CFI), acceptable if ≥ 0.90, Root Mean Square Error of Approximation (RMSEA), a good fit < 0.05, acceptable < 0.08 (Browne and Cudeck, 1992), and Standardized Root Mean Square Residual (SRMR), acceptable fit < 0.08 (Hu and Bentler, 1999).

#### 2.5.6 Reliability

Internal consistency was evaluated using Cronbach's alpha (α) and McDonald's omega (ω). Cronbach's alpha estimates internal consistency based on average inter-item correlation (Cronbach, 1951). According to conventional guidelines, α values > 0.70 are considered acceptable, values > 0.80 good, and values > 0.90 excellent (Nunnally and Bernstein, 1994).

McDonald's omega is a more robust alternative to alpha, particularly when the assumptions of tau-equivalence are violated. Omega is based on a factor-analytic model and is less sensitive to unequal item loadings and non-violated distributions (Dunn et al., 2014). Values between 0.70 and 0.90 are generally considered acceptable for psychological scales.

In addition, each item was examined through corrected item-total correlations (rit-c) to evaluate individual contribution to scale reliability. Items with a rit-c values < 0.30 indicate low item discrimination and limited contribution to the overall scale reliability.

#### 2.5.7 Validity

Convergent validity was assessed by examining associations between CEASY-SE subscales and theoretically related constructs. Divergent validity was evaluated through correlations with constructs expected to be unrelated or only weakly related to compassion. All correlations were calculated using Spearman's rank order correlations (*r*) (Schober et al., 2018). Correlation strength was interpreted using Cohen's guidelines (Cohen, 1988): *r* = 0.10 (small), *r* = 0.30 (moderate), and *r* ≥ 0.50 (strong).

Construct validity was assessed by comparing CEASY-SE scores between a clinical psychiatric sample and a normative school sample using independent samples *t*-tests to evaluate the scale's ability to distinguish between populations with different psychological profiles.

Criterion-related validity was evaluated using logistic regression analysis. The criterion variable was the presence of mild to severe depression (MADRS-S score > 12), and the predictor variable was the total Self-Compassion score from CEASY-SE. Odds ratios (ORs) were calculated to estimate the likelihood of depression symptoms at different levels of self-compassion.

## 3 Results

### 3.1 Sample characteristics

The final sample included 355 participants, of whom 287 were female (81%). Participants' ages ranged from 16 to 22 years (*M* = 18.12, *SD* = 2.08). Parental SES indicated that none were unemployed, students, or on sick leave. Detailed demographic information is presented in Table 1.

**Table 1:** Sample demographic and characteristics (*N* = 355).

**Characteristic**	**Category**	***n* (%)**	**Mean**	**SD**
Age (range 16–22)	16–17	195 (54.9)	18.12	2.08
	18–22	160 (45.1)		
Sex	Female	287 (80.8)		
	Male	68 (19.2)		
Born in Sweden	Yes	334 (94.1)		
Living situation	With both parents	157 (62.3)		
	With one parent	42 (16.7)		
	With one parent and new partner	24 (9.5)		
	Alone	3 (0.3)		
	Other	29 (11.2)		
	Missing	103		
Parental SES	Unskilled worker	25 (7.9)		
	Skilled worker	41 (12.9)		
	Assistant non-manual employee	23 (7.2)		
	Intermediate non-manual employee	105 (33.0)		
	Higher civil servant	94 (29.6)		
	Self-employed	30 (9.4)		
	Missing	37		
Contact with mental health care	Past year	103		
Diagnoses by professionals		123 (34.6)		
Diagnoses by patients		174 (49.0)		

SD, standard deviation. Valid percentages are reported.

### 3.2 Item analysis and scale distribution

Descriptive statistics for CEASY-SE items and subscales were computed (see Supplementary material), revealing that corrected item-total correlations (rit-c) were ≥ 0.30 across total and sex subscales, except for the item “I can stand my own different types of feelings” in the Self-Compassion Engagement subscale in the male sample (rit-c = 0.25). Despite this, the item was retained to preserve the conceptual breadth of the scale.

Tests of normality indicated that several CEASY-SE scales were significantly skewed (*p* < 0.05), particularly among females. The only subscale that met normality assumptions across both sexes was the Self-Compassion Engagement subscale (Shapiro-Wilk *p* = 0.526 for females, *p* = 0.246 for males; Kolmogorov-Smirnov *p* = 0.200 for both).

### 3.3 Sex and age differences in compassion

Descriptive statistics and group comparisons for CEASY-SE total and subscale scores by sex are presented in Table 2. Females scored significantly higher than males on the Compassion for Others total scale (*d* = 0.50, *p* < 0.001) as well as on the Engagement (*d* = 0.42, *p* = 0.002) and Action subscales (*d* = 0.50, *p* < 0.001). Conversely, males scored significantly higher than females on the Self-Compassion total scale (*d* = −0.41, *p* = 0.002), the Engagement (*d* = −0.33, *p* = 0.014), and Action subscales (*d* = −0.44, *p* = 0.001). No significant sex differences were found for the Compassion from Others total scale *(d* = −0.02, *p* = 0.878).

**Table 2:** Sex differences in CEASY-SE scales and subscales (*N* = 355).

**Scale/Subscale**	**Total (*N* = 355) M (SD)**	**Male (*n* = 68) M (SD)**	**Female (*n* = 287) M (SD)**	***t***	***P***	**Cohen's *d***	**Mann-Whitney *U***	**η^2^**
Self-compassion – total scale	47.29 (15.47)	52.38 (15.81)	46.09 (15.17)	−3.05	0.002	−0.41	1,789.00	0.02
Engagement	27.57 (8.68)	29.89 (8.25)	27.02 (8.70)	−2.47	0.014	−0.33	2,023.50	0.02
Action	19.73 (7.92)	22.49 (8.51)	19.07 (7.65)	−3.24	0.001	−0.44	1,898.00	0.03
Compassion from others – total scale	55.29 (17.02)	55.58 (15.72)	55.23 (17.34)	−0.15	0.878	−0.02	2,437.50	0.00
Engagement	29.60 (9.57)	30.20 (8.74)	29.46 (9.76)	−0.57	0.570	−0.08	2,298.00	0.00
Action	25.69 (8.48)	25.38 (8.09)	25.76 (8.58)	0.33	0.740	0.05	2,415.00	0.00
Compassion for others – total scale	75.90 (12.20)	71.10 (12.43)	77.04 (11.89)	3.67	< 0.001	0.50	1,987.00	0.04
Engagement	41.91 (7.07)	39.54 (7.18)	42.48 (6.94)	3.12	0.002	0.42	2,103.50	0.03
Action	33.99 (6.08)	31.56 (6.49)	34.56 (5.85)	3.73	< 0.001	0.50	1,956.00	0.04

All *p*-values are two-tailed. Cohen's d interpreted as small (≥0.20), medium (≥0.50), and large (≥0.80). Mann-Whitney *U* test was used when normality assumptions were violated. η^2^ (eta squared) interpreted as small (≥0.01), medium (≥0.06), and large (≥0.14).

To further examine the effects of both sex and age, a two-way ANOVA was conducted (see Table 3). Significant main effects of were found for the Compassion for Others total scale: *F*_(1, 351)_ = 13.74, *p* < 0.001, partial η^2^ = 0.04, and the Self-Compassion total scale, *F*_(1, 351)_ = 7.16, *p* = 0.008, partial η^2^ = 0.02 (see Table 3). Additionally, significant interaction effects between sex and age were observed for both Compassion for Other total scale, *F*_(1, 351)_ = 12.35, *p* < 0.001, partial η^2^ = 0.03, and the Self-Compassion total scale, *F*_(1, 351)_ = 6.39, *p* = 0.012, partial η^2^ = 0.02. Self-Compassion increased with age among females but decreased among males. A similar interaction pattern was observed for Compassion from Others, whereas Compassion for Others remained relatively stable across age groups. Age was analyzed as a categorical factor with two groups (16–17 and 18–22 years) to reflect development stages.

**Table 3:** Group differences in compassion: descriptive statistics and two-way ANOVA outcomes (*N* = 355).

**Scale**	**Age group 16–17 years M (SD)**	**Age group 18–22 years M (SD)**	**Effect**	***F* ratio**	***df***	***P***	**Partial η^2^**
Self-compassion- total scale	Females 42.66 (14.46)	49.90 (15.09)	Sex	7.16^**^	1,351	0.008	0.02
	Males 53.56 (17.90)	50.21 (11.03)	Age	0.86	1,351	0.355	0.00
			Sex^*^Age	6.39^*^	1,351	0.012	0.02
Compassion from other**s-**total scale	Females 51.47 (17.20)	59.39 (16.58)	Sex	0.22	1,351	0.639	0.00
	Males 58.56 (16.10)	50.13 (13.69)	Age	0.01	1,351	0.912	0.00
			Sex^*^Age	12.35^***^	1,351	< 0.001	0.03
Compassion for other**s-**total scale	Females 77.31 (12.53)	76.74 (11.18)	Sex	13.74^***^	1,351	< 0.001	0.04
	Males 71.83 (11.84)	69.75 (13.59)	Age	0.62	1,351	0.432	0.00
			Sex^*^Age	0.20	1,351	0.656	0.00

^*^p < 0.05.

^**^p < 0.01.

^***^p < 0.001.

M, mean; SD, standard deviation.

### 3.4 Compassion scale scores across psychiatric diagnoses

Table 4 presents descriptive statistics for total scales of Self-Compassion, Compassion for Others, and Compassion from Others across psychiatric diagnoses, based on combined clinician-assigned and self-reported diagnoses. As expected, participants with depression (*M* = 42.44, *SD* = 14.01) and eating disorders (*M* = 40.36, *SD* = 14.69) reported the lowest levels of Self-Compassion. In contrast, those with ADHD (*M* = 48.42, *SD* = 17.38) and autism (*M* = 49.00, *SD* = 17.04) showed relatively higher Self-Compassion scores. Compassion for Others was generally high across all groups, with the highest score in the depression group (*M* = 79.26, *SD* = 9.99). In contrast, Compassion from Others was lowest among participants with autism (*M* = 47.41, *SD* = 17.65). These findings suggest that compassion profiles vary across different diagnostic categories.

**Table 4:** Compassion scale scores across psychiatric diagnoses.

**Psychiatric diagnosis**	**Scale**	**N**	**Mean**	**SD**	**SE**	**Min**	**Max**
Various eating disorders	Self-compassion	14	40.36	14.69	3.93	17	66
	Compassion for others	14	80.0	6.85	1.83	70	88
	Compassion from others	14	54.29	16.13	4.31	24	79
Various anxiety disorders	Self-compassion	33	46.30	14.66	2.55	11	71
	Compassion for others	33	76.48	11.16	1.94	41	90
	Compassion from others	33	56.70	18.42	3.21	24	87
Various depressive disorders	Self-compassion	57	42.44	14.01	1.86	11	74
	Compassion for others	57	79.26	9.99	1.32	35	90
	Compassion from others	57	51.81	16.32	2.16	25	88
ADHD	Self-compassion	70	48.42	17.38	2.08	14	88
	Compassion for others	70	74.94	12.82	1.53	37	90
	Compassion from others	70	55.24	17.69	2.11	18	90
Autism	Self-compassion	17	49.0	17.04	4.13	18	84
	Compassion for others	17	64.59	18.75	4.55	30	86
	Compassion from others	17	47.41	17.65	4.28	20	74
Total	Self-compassion	211	45.86	15.77	1.09	11	88
	Compassion for others	211	76.12	12.84	0.88	30	90
	Compassion from others	211	53.68	17.11	1.19	18	90

Compassion scores are based on combined clinician-assigned and self-reported psychiatric diagnoses. The “Other” category was excluded due to heterogeneity and small sample size.

### 3.5 SES on the three CEASY-SE scales

No significant correlations were found between SES and any of the three CEASY-SE scales (all *p* > 0.05). A Chi-square test showed no significant differences in SES between sexes, χ^2^(5, *N* = 318) = 1.686, *p* = 0.891.

### 3.6 Factor structure

CFA supported the proposed two-factor structure for all three CEASY-SE scales:

***Model 1: Compassion for Others***

**Figure 1:**
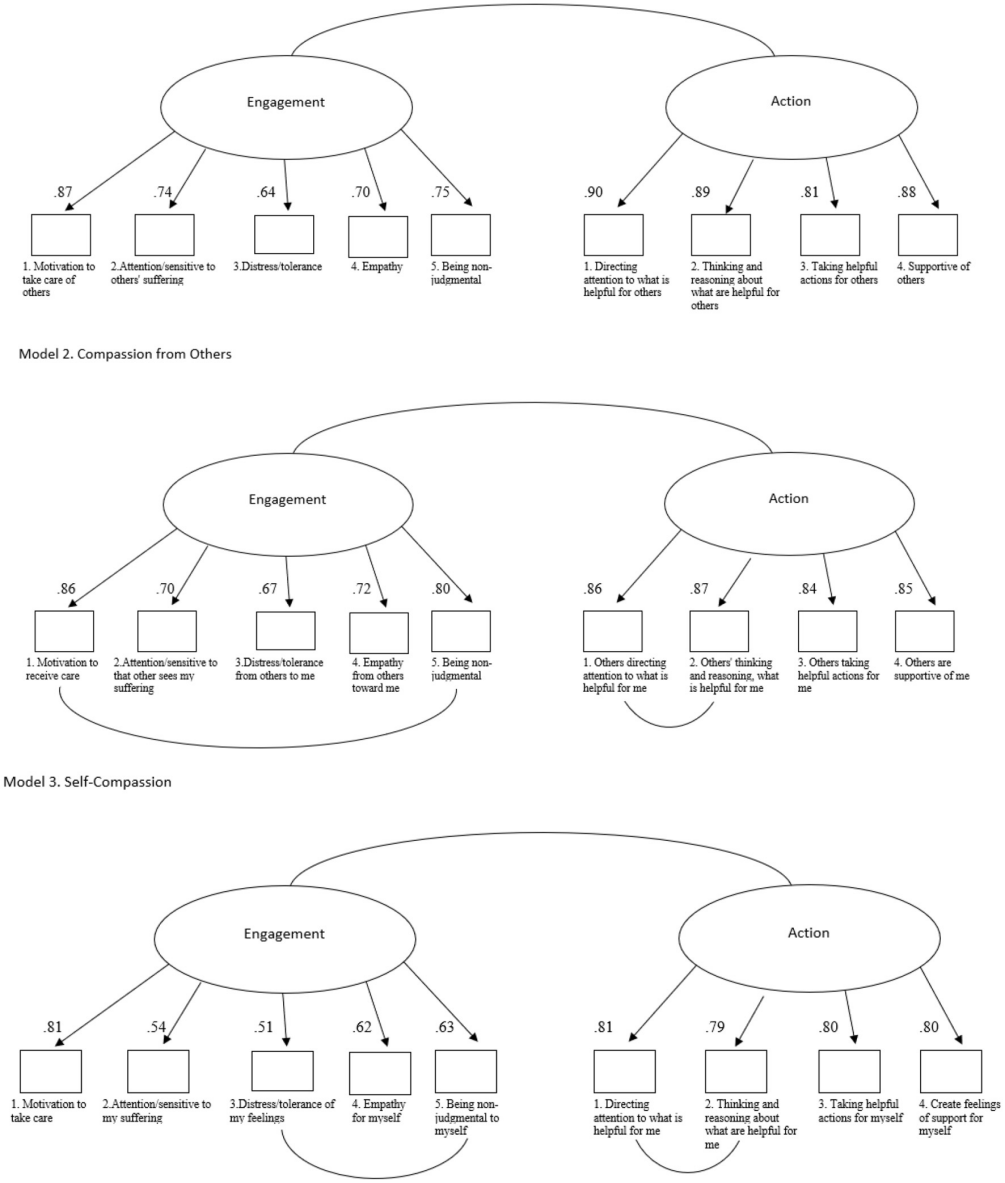
Graphical comparison of the three Confirmatory Factor Analysis; circles represent latent factors, and squares represent scale items.

The model demonstrated good fit (χ^2^(26) = 83.89, χ^2^*/df* = 3.22, CFI = 1.00, TLI = 0.99, RMSEA = 0.08 (90% CI [0.06, 1.00]), SRMR = 0.05), confirming the model's suitability. Standardized factor loadings exceeded 0.40 for all items (Figure 1, Model 1).

***Model 2: Compassion from Others***

The initial two-factor model showed poor fit (χ^2^(26) = 111.18, χ^2^/*df* = 4.28, CFI = 0.99, TLI = 0.99, RMSEA = 0.10 (90% CI [0.08, 0.12]), SRMR = 0.05). After allowing residual correlations between (1) item 1 and item 5 from the Engagement subscale and (2) item 1 and item 2 from the Action subscale, model fit improved (χ^2^(24) = 62.54, χ^2^*/df* = 2.61, CFI = 1.00, TLI = 1.00, RMSEA = 0.07 (90% CI [0.05, 0.09]), SRMR = 0.04). Factor loadings remained above 0.40 (see Figure 1, Model 2).

***Model 3: Self-Compassion***

The initial two-factor model showed suboptimal fit (χ^2^(26) = 103.02, χ^2^*/df* = 3.96, CFI = 0.99, TLI = 0.99, RMSEA = 0.09 (90% CI [0.07, 0.11]), SRMR = 0.05). After introducing residual correlations between (1) item 3 and item 5 from the Engagement subscale and (2) item 1 and item 2 from the Action subscale, model fit improved (χ^2^(24) = 62.81, χ^2^*/df* = 2.62, CFI = 1.00, TLI = 1.00, RMSEA = 0.07 (90% CI [0.05, 0.09]), SRMR = 0.04). All factor loadings were above 0.40 (see Figure 1, Model 3).

### 3.7 Reliability

The internal consistency of the CEASY-SE was generally good to excellent across all scales concerning the total sample, with Cronbach's alpha values ranging from 0.75 to 0.92 and McDonald's omega values showing similar strength. The only exception was the Self-Compassion Engagement subscale among males, which showed lower reliability (α = 0.62, ω = 0.60), suggesting potential variability in how males interpret or respond to these items. All other subscales demonstrated acceptable to excellent reliability, supporting the scale's robustness across most domains and subgroups. Table 5 summarizes the internal consistency estimates for each subscale, by sex, and for the total sample.

**Table 5:** Internal consistency of the CEASY-SE (*N* = 355).

**Scales**	**Males (*****n*** = **68)**		**Females (*****n*** = **287)**		**Total (*****N*** = **355)**	
	α	ω	α	ω	α	ω
Self-compassion- total scale	0.85	0.86	0.89	0.89	0.88	0.88
Engagement	0.62	0.60	0.78	0.77	0.75	0.75
Action	0.85	0.85	0.88	0.88	0.88	0.88
Compassion from others- total scale	0.89	0.89	0.93	0.93	0.92	0.92
Engagement	0.76	0.76	0.86	0.85	0.84	0.84
Action	0.90	0.92	0.92	0.90	0.91	0.92
Compassion for others- total scale	0.88	0.90	0.90	0.87	0.90	0.90
Engagement	0.77	0.76	0.81	0.82	0.81	0.81
Action	0.89	0.89	0.88	0.89	0.89	0.90

M, mean; SD, standard deviation; α, Cronbach alpha; ω, McDonald's omega.

Spearman's correlation analyses revealed strong internal relationships between Engagement and Action subscales within each compassion scale. For example, the correlation between Engagement and Action in the Compassion for Others scale was *r* = 0.65 (*p* < 0.01), indicating these components are closely related. In contrast, correlations between different scales (e.g., Self-Compassion and Compassion for Others) were weak or absent (*r* = 0.00–0.13), suggesting that these constructs are relatively independent in this sample. That is, being self-compassionate does not necessarily imply being more compassionate toward others, and vice versa. Table 6 presents the intercorrelations between the subscales.

**Table 6:** Intercorrelations between CEASY-SE subscales using Spearman's Rho (*N* = 355).

**Subscales**	**Self-compassion Engagement**	**Self-compassion Action**	**Compassion for others Engagement**	**Compassion for others Action**	**Compassion from others Engagement**	**Compassion from others Action**
Self-compassion Engagement	1.00	0.69^**^	0.13^*^	−0.02	0.32^**^	0.21^**^
Self-compassion Action	0.69^**^	1.00	0.05	0.00	0.35^**^	0.23^**^
Compassion for others Engagement	0.13^*^	0.05	1.00	0.65^**^	0.22^**^	0.23^**^
Compassion for others Action	−0.02	0.00	0.65^**^	1.00	0.16^**^	0.29^**^
Compassion from others Engagement	0.32^**^	0.35^**^	0.22^**^	0.16^**^	1.00	0.78^**^
Compassion from others Action	0.21^**^	0.23^**^	0.23^**^	0.29^**^	0.78^**^	1.00

^*^p < 0.05.

^**^p < 0.01.

### 3.8 Validity

#### 3.8.1 Convergent and divergent validity

Convergent validity was assessed by examining correlations between the CEASY-SE and theoretically related constructs: self-compassion (SCS), wellbeing (WHO-5), anxiety (RCADS Anxiety), and depression (RCADS Depression and MADRS-S). The CEASY-SE showed small to large positive correlations with self-compassion (*r* = 0.14 to 0.56, *p* < 0.01) and wellbeing (*r* = 0.12, *p* < 0.050, to 0.37, *p* < 0.010), indicating that higher CEASY-SE scores are associated with greater self-compassion and wellbeing.

Furthermore, the Self-Compassion and the Compassion from Others scales demonstrated negative correlations with anxiety and depression (*r* = −0.19 to −0.47, *p* < 0.010), supporting the protective role of compassion in mental health. Interestingly, the Compassion for Others scale showed small but significant positive correlations with anxiety and depression *(r* = 0.11 to 0.14, *p* < 0.05), suggesting that outward compassion may sometimes co-occur with internal psychological distress.

Divergent validity was supported by weak or non-significant correlations with unrelated constructions such as functional impairment (SDQ Impact; *r* = 0.05 to −0.21, *p* < 0.01) and physical activity (PROMIS: *r* = 0.00 to 0.12, *p* < 0.05), indicating that CEASY-SE might capture distinct psychological dimensions. See Table 7 for detailed correlation coefficients.

**Table 7:** Spearman's correlations between CEAS-Y-se subscales and validity measures (*N* = 355).

**Measure**	**SCS**	**WHO-5**	**RCADS anxiety**	**RCADS depression**	**MADRS-S depression**	**SDQ impact**	**PROMIS physical activity**
Self-compassion- total scale	0.56^**^	0.37^**^	−0.31^**^	−0.47^**^	−0.49^**^	−0.21^**^	0.12^*^
Compassion for others- total scale	0.14^**^	0.12^*^	0.14^**^	0.11^*^	0.12^*^	0.05	0.00
Compassion from others- total scale	0.22^**^	0.28^**^	−0.19^**^	−0.29^**^	−0.31^**^	−0.21^**^	0.09

^*^p < 0.05.

^**^p < 0.01.

SCS, self-compassion scale; WHO-5, WHO wellbeing index; RCADS, the revised child anxiety and depression scale; MADRS-S, Montgomery Åsberg depression rating scale; SDQ impact, strength and difficulties questionnaire - functioning composite subscale; PROMIS physical activity, patient reported outcome measurements information system item bank PROMIS pediatric physical activity v1.0.

#### 3.8.2 Construct validity

Construct validity was examined by comparing the CEASY-SE scores between the clinical psychiatric sample and the school sample using independent *t*-tests. The clinical psychiatric sample scored lower than the school sample on the Self-Compassion scale (*d* = −0.56), indicating a moderate effect size. Similarly, the clinical group reported lower scores on the Compassion from Others scale (*d* = −0.29), reflecting a small effect size. In contrast, the clinical sample scored slightly higher on the Compassion for Others scale (*d* = 0.20), also a small effect size. These findings suggest that while young people may demonstrate outward compassion, they often experience less compassion from others and toward themselves. This supports the CEASY-SE's ability to differentiate between populations with varying psychological profiles and levels of wellbeing. See Table 8 for more details.

**Table 8:** Independent *t*-test comparing clinical (*N* = 355) and school (*N* = 316) samples on CEASY-SE subscales.

**Scales**	**Clinical sample** ***N*** = **355**		**School sample** ***N*** = **316**		**Independent** ***t*****-test**		
	**M**	**SD**	**M**	**SD**	***t***	***P***	**ES** ***d***
Self-compassion-total scale	47.29	15.47	56.23	16.34	−7.27	< 0.001	−0.56
Compassion from others- total scale	55.29	17.02	60.02	16.06	−3.69	< 0.001	−0.29
Compassion for others- total scale	75.90	12.20	73.36	13.06	2.61	0.009	0.20

M, mean; SD, standard deviation; ES, effect size (Cohen's *d*). *d* ≥ 0.20 = small, ≥ 0.50 = moderate, ≥ 0.80 = large effect.

#### 3.8.3 Criterion-related validity

Criterion-related validity, was assessed by examining the predictive value of the Self-Compassion scale scores on depression symptoms, defined as >12 points on MADRS-S/indicating more than mild symptoms of depression. A binary logistic regression showed that the model was statistically significant (χ^2^(1, *N* = 355) = 97.75, *p* < 0.001), explained 26.0% of the variance in depression symptoms (Nagelkerke *R*^2^ = 0.26), and correctly classified 80.3% of cases. This indicates that the Self-Compassion scale scores meaningfully predict depression symptoms.

Lower Self-Compassion scores were associated with a substantially increased risk of depressive symptoms. For instance, a total Self Compassion score of 40 or lower was associated with an 8.4–fold increase in the odds of depression compared to a score of 100. Similarly, scores in the 20–40 range were linked to a high probability of depression symptoms (e.g., 89–97%). Odds ratios and expected probabilities are presented in Table 9.

**Table 9:** Odds ratios and expected probabilities for self-compassion scores predicting depression symptoms (*N* = 355).

**Value on the self-compassion scale**	**Expected probability**	**Odds ratio**	**Risk of depression symptoms**
20	0.97	38.47	Likely
40	0.89	8.41	Possibly
60	0.65	1.84	Less Possibly
80	0.29	0.40	Unlikely

Expected probability refers to the likelihood of depression symptoms based on self-compassion scores. Odds ratio indicates the increase or decrease in odds of depression symptoms compared to a reference score (e.g., 100). Risk categories are interpretative and based on relative odds.

## 4 Discussion

This is the first study to validate the CEASY-SE in a clinical psychiatric population of youth. While previous validations have focused on school-based or community samples (Henje et al., 2020; Cunha et al., 2023), the current study addresses a gap by evaluating the scale in a clinical setting. The findings indicate that the CEASY-SE demonstrates acceptable to strong psychometric properties in this context, including a replicable factor structure, generally good internal consistency, and evidence of various forms of validity. The overall results suggest that the CEASY-SE is a promising tool for assessing compassion in youth mental health care. These findings are consistent with Gilbert's (Gilbert et al., 2017) theoretical framework of compassion and extend previous studies conducted in school-based and international samples (Henje et al., 2020; Cunha et al., 2023).

### 4.1 Factor structure

CFA supported the proposed two-factor structure for each of the three CEASY-SE scales. While the Compassion for Others scales showed good model fit without modifications, the Compassion from Others and Self-Compassion scales required minor adjustments (residual correlations) to improve fit. These findings align with previous validations of the CEAS and CEASY-SE both in young and adult populations (Cunha et al., 2023; Gilbert et al., 2017; Henje et al., 2020). The need for model refinement highlights the complexity of measuring compassion, particularly in clinical youth populations, and suggests that further refinement may enhance model fit in future studies.

### 4.2 Reliability

Reliability, in terms of internal consistency, was good to excellent across most scales and subscales except the Self-Compassion Engagement scale among males. This mirrors findings from earlier studies and suggests that males may interpret or respond to self-compassion differently (Cunha et al., 2023; Gilbert et al., 2017; Henje et al., 2020). This discrepancy should be taken into account when interpreting the results for this subgroup and may warrant refinement of the scale.

The strong correlations within the Compassion for Others, Compassion from Others, and Self-Compassion scales support the internal consistency of the scales. Specifically, the strong correlations between the Engagement and Action subscales within each category suggest that these aspects of compassion are closely related. In contrast, the weak correlation between Self-Compassion and Compassion for Others subscale indicates that these are relatively independent constructs. These support the theoretical distinction between the three flows of compassion and underscore the importance of assessing them separately.

### 4.3 Validity

As expected, CEASY-SE scores were positively associated with self-compassion and wellbeing and negatively associated with anxiety and depression. These findings support the scale's convergent validity and reinforce the protective role of compassion in mental health. Interestingly, the Compassion for Others scale showed small positive correlations with anxiety and depression, which may reflect emotional burden or empathic distress in highly compassionate individuals, a pattern also observed in previous CEAS research (Gilbert et al., 2017; Brophy et al., 2024). This finding suggests that outward compassion, although generally adaptive, may be associated with emotional strain in vulnerable populations.

Divergent validity was supported by weak or non-significant correlations with unrelated constructs, such as physical activity and functional impairment, indicating that the CEASY-SE measures distinguish between psychological domains. This strengthens the argument that the scale captures specific compassion-related constructs rather than general distress or functioning. This result aligns with earlier studies (Brophy et al., 2024; Gilbert et al., 2017). Similarly, the initial validation study of the CEASY-SE found low correlations with unrelated constructs, supporting the divergent validity of the scales (Henje et al., 2020).

The CEASY-SE effectively distinguished between clinical and non-clinical youth. While clinical participants reported higher on the Compassion for Others scale, they scored significantly lower on the Self-Compassion and Compassion from Others scales. These findings suggest that although young people in psychiatric care may be attuned to others' suffering, they struggle more with receiving and directing compassion inward. This pattern is consistent with clinical observations and empirical findings showing that self-criticism and shame are common among youth with internalizing disorders (Mcintyre et al., 2018). It underscores the importance of targeting self-compassion and receptivity to support in therapeutic interventions for young people in clinical psychiatric care.

Additionally, scores on the Self-Compassion scale varied across different psychiatric diagnoses. As expected, the lowest levels were observed among participants with depression and eating disorders, which are conditions commonly associated with high self-criticism and shame. In contrast, participants with autism and ADHD reported relatively higher self-compassion. These differences may reflect diagnostic-specific emotional processing styles, such as reduced self-monitoring in ADHD or unique cognitive-emotional processing in autism. However, these interpretations remain speculative and require further research (Cai et al., 2023). Scores on the Compassion for Others scale were generally high across all groups, with the highest scores in the depression group, which possibly reflects heightened sensitivity to others' suffering. Meanwhile, scores on the Compassion from Others scale were lowest among participants with autism, potentially indicating difficulties in perceiving or receiving social support. However, this shows the potential value of tailoring compassion-based interventions to specific diagnostic groups, particularly those with internalizing disorders.

These findings are consistent with Gilbert's theoretical model, which suggests that the three flows of compassion, self-compassion, compassion for others, and compassion from others, are only moderately correlated and may function independently (Gilbert et al., 2017; Gilbert, 2017). For instance, an individual may be highly self-compassionate but not necessarily receive much compassion from others. This aligns with our findings, which showed that while the Self-Compassion scale was strongly correlated with mental health outcomes, Compassion for Others and Compassion from Others scales had more varied relationships. These distinctions highlight the importance of evaluating each compassion flow independently in both research and clinical practice.

Self-Compassion scores significantly predict the likelihood of depressive symptoms. The logistic regression model explained 26% of the variance in depression symptoms, correctly classifying 80.3 % of cases. This suggests that self-compassion may serve as a clinically meaningful indicator of vulnerability in youth. Previous studies have consistently shown that higher levels of self-compassion are associated with lower levels of negative emotional states (Gilbert, 2020; Marsh et al., 2018). These findings confirm the clinical relevance of self-compassion and support its use as a target for screening or intervention in youth mental health care. It also supports the use of CEASY-SE in clinical settings to assess and enhance self-compassion as a protective factor against mental health issues.

### 4.4 Sex and age differences in compassion

Our findings revealed sex and age differences in compassion, as well as developmental patterns. Females reported higher levels on the Compassion for Others scale, while males scored higher on the Self-Compassion scale. Given the predominance of female participants, the observed sex differences should be interpreted with caution, as the smaller number of male participants may have influenced variability and statistical power. However, these results are consistent with previous research in normative samples (Henje et al., 2020; Cunha et al., 2023), suggesting that females may be more attuned to the needs of others, potentially at the expense of their own self-compassion. This aligns with the broader literature, which indicates that females often exhibit higher levels of empathy and caregiving behaviors (Pang et al., 2023).

We observed a sex-by-age interaction: self-compassion increased with age among females but decreased among males. This pattern diverges from previous findings by Bluth et al. (2017), who reported that self-compassion tends to decline with age among adolescent girls, while remaining relatively stable among boys. One possible explanation for this discrepancy is that our sample included older adolescents and young adults (16–22 years), whereas Bluth's study (Bluth et al., 2017) focused on a younger adolescent (14–17 years). Differences between clinical and community samples may also influence the development of compassion across different age groups.

In this study, age was analyzed using two predefined categories: adolescents (16–17 years) and young adults (18–22 years), reflecting key developmental stages. This approach enabled us to investigate whether compassion-related processes vary between late adolescence and early adulthood. The observed interaction may reflect distinct developmental trajectories, where older females benefit from greater emotional regulation and self-reflection, whereas males might be more influenced by social or cultural factors that inhibit self-compassion. Future studies could build on these findings by combining categorical and continuous age analyses to capture more nuanced developmental patterns.

These findings suggest that the ability to give and receive compassion develops differently across age groups in clinical populations. Developmental stages present unique challenges: younger people often focus on identification and social relationships, while older people develop greater emotional regulation and self-reflection through more life experiences and emotional resilience (Tavares et al., 2024). Tailoring interventions to address these sex and age differences could improve their effectiveness. For instance, programs for females might focus on building self-compassion, while those for males could emphasize the importance of receiving compassion from others. Future research should continue to explore these patterns in diverse clinical and non-clinical populations.

This highlights the importance of adopting a multidisciplinary perspective when studying compassion in youth. Developmental psychology, cultural psychology, and evolutionary theory each offer complementary insights into how compassion emerges and functions across contexts. Evolutionary models emphasize caregiving systems and social affiliation as foundational mechanisms (Gilbert, 2017), whereas cultural frameworks point to variations in how compassion is expressed, valued, and internalized across societies (Jiang et al., 2023; Strauss et al., 2016). Adolescence is a particularly dynamic period in which identity formation intersects with cultural norms, gender roles, and social expectations, all of which may shape the development and expression of compassion. Integrating these perspectives can enhance our understanding of compassion as both a universal and culturally embedded phenomenon and may inform the generalizability of findings beyond the Swedish clinical context.

### 4.5 Clinical implications

CEASY-SE is a promising tool for assessing compassion in clinical youth populations. It can help clinicians identify compassion-related strengths and vulnerabilities, guide treatment planning, and monitor therapeutic progress. Its multidimensional structure enables a nuanced understanding of how compassion is experienced and expressed across various relational contexts. Given the strong link between self-compassion and mental health, compassion-focused interventions (e.g., CFT) may be particularly beneficial for this group. Future studies should also explore the scale's sensitivity to change in response to intervention to further establish its clinical utility.

### 4.6 Limitations

Several limitations should be noted. First, the sample was drawn from clinics in northern Sweden and was predominantly female, which may limit generalizability. Second, the cross-sectional design precludes causal inferences. Third, the reliance on self-report measures introduces potential biases such as social desirability. Finally, although the sample encompassed a range of psychiatric diagnoses, subgroup sizes were small, which limited the ability to draw specific conclusions for each diagnosis.

### 4.7 Future research

Future research on CEASY-SE should aim to replicate these findings in a more diverse and balanced sample, including younger adolescents and underrepresented sexes. Studies should also focus on specific psychiatric diagnoses to better understand the tool's applicability. Longitudinal studies are necessary to investigate how compassion evolves over time and in response to treatment. Further refinement of the Self-Compassion Engagement subscale for males may also be warranted. Additionally, exploring the scale's sensitivity to change in intervention studies would strengthen its utility in clinical practice.

### 4.8 Conclusions

This study aimed to validate the psychometric properties CEASY-SE in a clinical psychiatric sample of young people. The findings demonstrate that CEASY-SE is a reliable and valid instrument for assessing compassion across three relational flows—toward others, from others, and toward oneself. Despite some limitations, the scale showed good to excellent internal consistency, a replicable factor structure, and substantial evidence of convergent, divergent, construct, and criterion-related validity.

In addition to confirming the scale's psychometric soundness, this study contributes novel insights into how compassion varies across psychiatric diagnoses and interacts with age and sex. Notably, self-compassion was found to decrease with age among males, while increasing among females, highlighting the need for sex-sensitive interventions. Furthermore, the substantial predictive value of self-compassion for depressive symptoms underscores its clinical relevance as a screening and intervention target.

## Data Availability

The raw data supporting the conclusions of this article will be made available by the authors, without undue reservation.
