# Supplementary material for: The Compassionate Engagement and Action Scale for Youths: psychometric properties in a clinical psychiatric Swedish sample
Source: Front Psychol. 2026 Jan 5;16:1653979. doi: 10.3389/fpsyg.2025.1653979 (PMC12812922; doi:10.3389/fpsyg.2025.1653979)
Supplement: Supplementary file 1 [file Supplementary_file_1.docx]

Supplementary Material

1. *Descriptive Statistics for Items in the Total Sample, Males and Females in the Engagement and Action Subscales of Compassion for Others, Compassion from Others and Self-Compassion*

|  | Total sample  N = 355 | | | | Males  N = 68 | | | | Females  N = 287 | | | |
| --- | --- | --- | --- | --- | --- | --- | --- | --- | --- | --- | --- | --- |
| Items | M | SD | *r*_it_ ^c^ | *α-i* | M | SD | *r*_it_ ^c^ | *α-i* | M | SD | *r*_it_ ^c^ | *α-i* |
| **Engagement subscale**  Compassion for Others: When others are distressed or upset by things… |  |  |  |  |  |  |  |  |  |  |  |  |
| Compassion from Others: When I’m distressed or upset by things… |  |  |  |  |  |  |  |  |  |  |  |  |
| Self-Compassion: When I’m distressed or upset by things… |  |  |  |  |  |  |  |  |  |  |  |  |
| 1a… I want to help others to feel better | 9.03 | 1.57 | 0.68 | 0.75 | 8.46 | 1.94 | 0.63 | 0.70 | 9.16 | 1.44 | 0.70 | 0.76 |
| 1b…others want to help me to feel better | 6.68 | 2.48 | 0.66 | 0.80 | 6.69 | 2.47 | 0.59 | 0.69 | 6.68 | 2.49 | 0.67 | 0.82 |
| 1c… I want to help myself to feel better | 5.84 | 2.47 | 0.53 | 0.70 | 6.68 | 2.72 | 0.45 | 0.52 | 5.64 | 2.37 | 0.55 | 0.74 |
| 2a…I notice the feelings of others | 8.54 | 1.66 | 0.56 | 0.79 | 8.03 | 1.86 | 0.60 | 0.71 | 8.66 | 1.58 | 0.53 | 0.80 |
| 2b…others notice my feelings | 5.54 | 2.42 | 0.63 | 0.81 | 5.51 | 2.45 | 0.54 | 0.71 | 5.54 | 2.42 | 0.65 | 0.83 |
| 2c…I notice my own feelings | 6.66 | 2.49 | 0.47 | 0.73 | 6.51 | 2.66 | 0.37 | 0.56 | 6.69 | 2.45 | 0.51 | 0.75 |
| 3a….I can stand their different types of feelings | 7.77 | 2.26 | 0.57 | 0.79 | 7.51 | 2.19 | 0.58 | 0.72 | 7.83 | 2.27 | 0.57 | 0.80 |
| 3b….others can stand different types of my feelings | 5.87 | 2.42 | 0.60 | 0.82 | 6.09 | 2.43 | 0.34 | 0.78 | 5.82 | 2.42 | 0.66 | 0.83 |
| 3c…I can stand my own different types of feelings | 5.24 | 2.47 | 0.44 | 0.74 | 5.81 | 2.66 | 0.25 | 0.63 | 5.10 | 2.41 | 0.49 | 0.76 |
| 4a…I can understand their feelings | 7.88 | 2.05 | 0.61 | 0.77 | 7.44 | 2.05 | 0.43 | 0.77 | 7.99 | 2.04 | 0.65 | 0.76 |
| 4b…others can understand my feelings | 5.00 | 2.53 | 0.67 | 0.80 | 5.30 | 2.51 | 0.60 | 0.69 | 4.92 | 2.53 | 0.69 | 0.82 |
| 4c…I can understand my feelings | 4.96 | 2.38 | 0.55 | 0.70 | 5.50 | 2.51 | 0.48 | 0.51 | 4.84 | 2.33 | 0.57 | 0.73 |
| 5a…I accept their feelings | 8.70 | 1.76 | 0.62 | 0.77 | 8.10 | 1.88 | 0.48 | 0.75 | 8.84 | 1.70 | 0.64 | 0.77 |
| 5b…others accept my feelings | 6.52 | 2.40 | 0.66 | 0.80 | 6.60 | 2.38 | 0.58 | 0.70 | 6.50 | 2.41 | 0.68 | 0.82 |
| 5c…I accept my feelings | 4.87 | 2.43 | 0.60 | 0.68 | 5.40 | 2.55 | 0.32 | 0.59 | 4.75 | 2.39 | 0.66 | 0.70 |
| **Action**  Compassion for Others: When others are distressed or upset by things… |  |  |  |  |  |  |  |  |  |  |  |  |
| Compassion from Others: When I’m distressed or upset by things… |  |  |  |  |  |  |  |  |  |  |  |  |
| Self-Compassion: When I’m distressed or upset by things… |  |  |  |  |  |  |  |  |  |  |  |  |
| 1a…I focus my attention on things that can help them | 8.55 | 1.68 | 0.81 | 0.84 | 7.81 | 1.77 | 0.80 | 0.84 | 8.72 | 1.61 | 0.80 | 0.83 |
| 1b…others focus their attention on things that can help me | 6.21 | 2.42 | 0.84 | 0.88 | 6.09 | 2.34 | 0.79 | 0.88 | 6.24 | 2.45 | 0.85 | 0.88 |
| 1c…I focus my attention on things that can help me | 5.19 | 2.32 | 0.78 | 0.82 | 5.91 | 2.56 | 0.78 | 0.78 | 5.02 | 2.23 | 0.78 | 0.83 |
| 2a….I find ways to help them handle their feelings | 8.49 | 1.89 | 0.80 | 0.84 | 7.88 | 1.84 | 0.77 | 0.85 | 8.63 | 1.87 | 0.80 | 0.83 |
| 2b…others will find ways to help me handle my feelings | 6.09 | 2.54 | 0.86 | 0.87 | 6.13 | 2.36 | 0.85 | 0.85 | 6.08 | 2.59 | 0.86 | 0.87 |
| 2c…I find ways to handle my feelings | 5.22 | 2.47 | 0.76 | 0.83 | 5.63 | 2.55 | 0.71 | 0.81 | 5.12 | 2.44 | 0.77 | 0.83 |
| 3a…I do things that will help them to feel better | 7.92 | 1.92 | 0.75 | 0.86 | 7.47 | 2.08 | 0.73 | 0.87 | 8.02 | 1.87 | 0.76 | 0.85 |
| 3b….others do things that will help me to feel better | 6.03 | 2.29 | 0.77 | 0.90 | 6.00 | 2.37 | 0.75 | 0.89 | 6.03 | 2.28 | 0.78 | 0.90 |
| 3c…I do things that will help me to feel better. | 5.32 | 2.21 | 0.73 | 0.84 | 6.07 | 2.36 | 0.63 | 0.84 | 5.14 | 2.14 | 0.75 | 0.84 |
| 4a…I am kind and supportive to them | 9.03 | 1.50 | 0.69 | 0.88 | 8.40 | 1.80 | 0.73 | 0.86 | 9.18 | 1.38 | 0.67 | 0.89 |
| 4b…others are kind and supportive to me | 7.36 | 2.24 | 0.75 | 0.91 | 7.16 | 2.10 | 0.76 | 0.89 | 7.41 | 2.27 | 0.75 | 0.91 |
| 4c…I am kind and supportive to myself | 4.00 | 2.28 | 0.67 | 0.87 | 4.87 | 2.73 | 0.68 | 0.82 | 3.79 | 2.12 | 0.66 | 0.87 |

*Note*. M = mean, SD = standard deviation, r_it_ ^c^ = corrected item-total correlation, α-i = ordinal alpha if the item is removed.
